# Shrimp Protein Hydrolysate Modulates the Timing of Proinflammatory Macrophages in Bupivacaine-Injured Skeletal Muscles in Rats

**DOI:** 10.1155/2016/5214561

**Published:** 2016-10-27

**Authors:** Junio Dort, Nadine Leblanc, Piotr Bryl, Marie-Gil Fortin, Marie-Elise Carbonneau, Charles Lavigne, Hélène Jacques

**Affiliations:** ^1^School of Nutrition, Laval University, Quebec City, QC, Canada G1V 0A6; ^2^Institute of Nutraceuticals and Functional Foods, Laval University, Quebec City, QC, Canada G1V 0A6; ^3^Merinov, Quebec Fisheries and Aquaculture Innovation Centre, 96 montée de Sandy Beach, Office 1.07, Gaspé, QC, Canada G4X 2V6; ^4^ISMER, Université du Québec à Rimouski, 300 allée des Ursulines, P.O. Box 3300, Rimouski, QC, Canada G5L 3A1; ^5^Quebec Agrifood Development Center, La Pocatière, QC, Canada

## Abstract

This study was designed to determine whether marine-derived proteins other than cod could have beneficial effects on inflammation following muscle injury. Macrophage and neutrophil densities were measured from bupivacaine-injured tibialis anterior muscle of rats fed isoenergetic diets containing either shrimp hydrolysate (Shr), casein hydrolysate (CaH), or whole casein (Ca). In this study, Shr reduced ED^1+^-macrophages at day 2 (*p* = 0.013), day 5 (*p* = 0.006), and day 14 after injury (*p* = 0.038) compared with Ca, indicating faster resolution of inflammation in Shr. Except for day 2 after injury where Shr led to lower ED^1+^-macrophages compared with CaH (*p* = 0.006), both Shr and CaH responded similarly at days 5, 14, and 28 after injury. This findings suggest that beneficial effects of Shr on ED^1+^-cells might be related to generation of anti-inflammatory peptides through the hydrolysis process, in addition to its high content of anti-inflammatory amino acids. However, while increasing myofiber cross-sectional area in noninjured muscles compared with both Ca and CaH, Shr failed to have a positive effect in corresponding injured muscles. These data indicate that shrimp hydrolysate can facilitate resolution of inflammation after muscle injury mainly through modulating proinflammatory macrophage accumulation but have less effect on optimal recovery in terms of muscle mass and fiber size.

## 1. Introduction

Skeletal muscle injury is a common clinical issue that can be caused by several conditions including direct trauma, prolonged training, ischemia, or myotoxins. Repair of damaged fibers is a tightly regulated process, consisting of an early inflammatory response, which dictates muscle protein breakdown, myogenic specification, and differentiation; regeneration of skeletal muscle is involved in direct relation to the timing of inflammation [1, 2]. Besides conventional use of pharmaceutical tools that are also being studied, protein feeding is a newly exciting approach to modulating injury-induced inflammation through providing amino acids that can induce a generalized beneficial effect on muscle regeneration [3].

Neutrophils and ED1^+^-macrophages are first myeloid cells that invade skeletal muscle at the onset of injury-induced inflammation in rats [4]. They are typically associated with removal of tissue debris, which is followed by the accumulation of the anti-inflammatory ED2^+^-macrophage subtype [4, 5]. Although phagocytosis of debris appears to be critical for initiation of muscle regeneration, it is believed that excessive influx of either neutrophils or ED1^+^-macrophages can induce a more complex inflammatory response that can have long-term negative effects on muscle repair [4]. Studies in mice have evaluated the participation of either neutrophils or phagocytic macrophages up to 38% or 80% in chronic muscle injuries, respectively [6, 7], indicating that modest depletion of neutrophils and ED1^+^-macrophages can reduce muscle damage, thereby accelerating repair. In contrast, ED2^+^-cells are associated with wound healing, owing to their ability to induce the myogenic lineage and remodeling, as well as modulating ED1^+^-related cytotoxicity [4]. There is thus a basic similarity between insufficient and exacerbated inflammation in impairing muscle regeneration, while the latter is more commonly observed during acute muscle injuries.

Marine-derived proteins including fish and crustaceans are high-quality proteins that have been shown to exert potential health benefits ranging between antihypertensive, antioxidant, antimicrobial, anticoagulant, antidiabetic, hypocholesterolemic [8], and anti-inflammatory activities [3, 9–11]. More importantly, our laboratory has recently revealed the effectiveness of dietary fish protein at enhancing growth and regeneration of skeletal muscle after trauma, through decreasing proinflammatory ED1^+^-cells [3, 9] and increasing anti-inflammatory ED2^+^-cells [3] as well as activating IGF1-Akt/PKB signaling pathway in rat skeletal muscle during recovery from injury [12]. Further strong evidences have shown that high levels of anti-inflammatory amino acids arginine, glycine, and taurine might account for beneficial effects of cod protein on inflammatory cell accumulation after injury [3], suggesting that dietary proteins other than cod rich in these amino acids might have similar modulatory effects on injury-induced inflammation.

The present study investigated the impact of shrimp protein hydrolysate (Shr) compared with casein hydrolysate (CaH) and whole casein (Ca), as controls, on the timing of inflammation cells in chemically injured skeletal muscle in rats. Shrimp protein is a high-quality protein source with elevated amounts of arginine, glycine, and taurine [13], which have been consistently shown to modulate inflammation in various rodent models of inflammation [14–18]. Our working hypothesis was that shrimp protein hydrolysate beneficially modulates the time course of inflammation following muscle injury due to its high content of anti-inflammatory amino acids arginine and glycine. We further performed morphometric analyses (muscle mass, cross-sectional area, and central nucleation) along with myogenic marker measurements (myoD and myogenin) in injured tibialis anterior muscle to quantify muscle regeneration.

## 2. Materials and Methods

### 2.1. Ethics Statement

This study was approved by the Animal Care Committee at Laval University (Quebec, permit number 2012155-1) in strict accordance with the Canadian Council on Animal Care guidelines. Buprenorphine (0.1 mg/kg) was administrated before anesthetizing animals with inhaled isoflurane (2.5%/L O_2_). No adverse effects were observed in locomotion or health status.

### 2.2. Animals and Experimental Design

**Figure 1:**
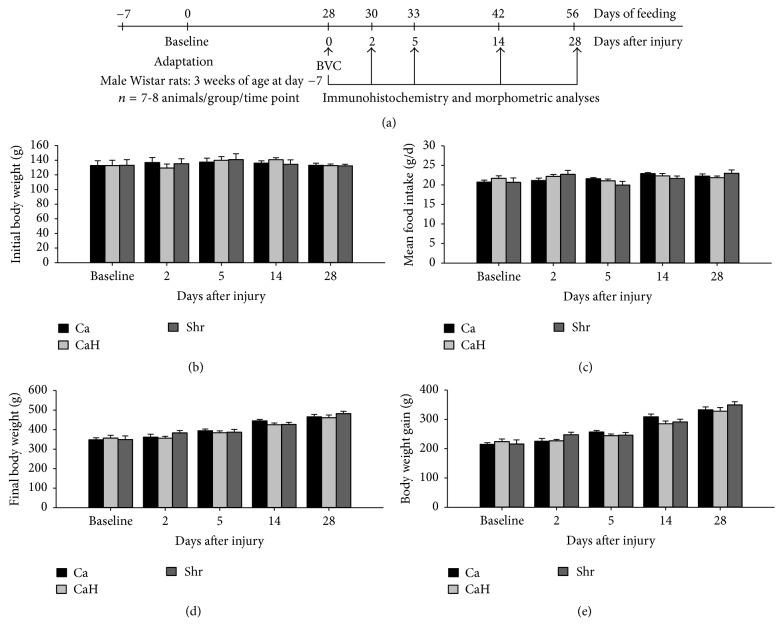
Effect of dietary proteins on zootechnical parameters of rats after muscle injury. Rats were fed diets for 56 days (including 28 days before injury and 28 days after injury), containing either Ca, CaH, or Shr. Timeline of sacrifice and tibialis anterior muscle collection after bupivacaine injection (BPV) is shown in (a). Initial body weight (b), mean food intake (c), final body weight (d), and body weight gain (e) are given for each studied time point. Values are mean ± SEM (*n* = 7-8 rats per group/time point). Ca, casein; CaH, casein hydrolysate; Shr, shrimp protein hydrolysate.

One hundred and twenty male Wistar rats (Charles River, Saint-Constant, Quebec, Canada), approximately 3 weeks of age, were housed individually in plastic cages maintained at 20°C (45–55% of humidity) on a 12 : 12 h light : dark cycle in specific pathogen-free conditions at the animal holding facility (INAF, Laval University, Quebec, Canada). Following 7-day adaptation provided by feeding a ground nonpurified diet (NPD) (Rodent Chow, Ralston Purina Inc., Quebec, Canada), animals were randomly assigned to one of three experimental diets, which differ in protein source (*n* = 40 rats per dietary group). They were then progressively transferred to their respective experimental purified diet (ED) over a 4-day period (100% NPD for 1 day; 25% ED and 75% NPD for 1 day; 50% ED and 50% NPD for 1 day; and 75% ED and 25% NPD for 1 day). Each of the three experimental groups was divided into five subgroups according to the day of sacrifice (0, 2, 5, 14, and 28 after injury) (Figure 1(a)). Water and food were provided* ad libitum* throughout the experimental period (including 28 days before injury and 28 days after injury). Food intake and body weight were recorded every two days.

### 2.3. Experimental Purified Diets

Powdered purified diets were formulated and prepared in our laboratory and contained 20% of protein, consisting of either casein (Ca, 89.7% protein), casein hydrolysate (CaH, 89.7% protein), or shrimp hydrolysate (Shr, 93.9% protein). The amino acid composition of each protein source and the formulation of ED are given in Tables 1 and 2, respectively. Shr hydrolysate was produced at Merinov Centre (Quebec, Canada) on a pilot scale. Briefly, minced meat of northern shrimp (*Pandalus borealis*) was mechanically extracted from by-products of shrimp processing plants. Enzymatic hydrolysis of minced meat was performed with a food grade protease (Novozymes, Bagsværd, Denmark). Soluble proteins contained in hydrolysates were separated from oil and other insoluble matters by centrifugation and protein hydrolysates were further fractionated by ultrafiltration and nanofiltration. All other ingredients were supplied by MP Biomedicals (Solon, Ohio, USA), except for lard and soybean oil, which were purchased from local supermarkets. To minimize oxidation of either n-6 (PUFA) in lard or n-6 and n-3 (PUFA) in soybean oil, butylated hydroxytoluene (BHT) was added to ED. Soybean oil was added to meet essential fatty acid requirement of rats [19]. The level of protein in ED was adjusted to an isonitrogenous basis at the expense of cornstarch and sucrose.

ED were formulated to be isoenergetic, isolipidic, and isonitrogenous. As expected, the energy content measured by automatic adiabatic calorimeter (model 1241; Parr Instruments, Moline, Illinois, USA) was similar between diets (Ca, 4.90 kcal/g; CaH, 4.93 kcal/g; Shr, 4.83 kcal/g). The protein content (N × 6.25) determined by Dumas method (Leco FP-528, ISO 34/SC 5, Ontario, Canada) was also similar between ED (Ca, 20.4%; CaH, 19.6%; Shr, 20.8%). In addition, no difference was found in the lipid content measured by an extraction method (Ankom^XT10^ Extractor, Ankom Technology, Macedon, New York, USA) between ED (Ca, 14.0% (W/W); CaH, 13.4% (W/W); Shr, 15.1% (W/W)).

### 2.4. Amino Acid Analysis

The determination of amino acids, except tryptophan, was made using the AccQ-Tag amino acid analysis procedure (Waters, Mississauga, ON, Canada), a precolumn derivatization technique for determination of total amino acids [20]. After acidic or basic hydrolysis, all amino acids were separated by reversed-phase high performance liquid chromatography (RP-HPLC) and quantified by fluorescence detection, using previously described conditions [20]. A Water Alliance Separations module e2695 equipped with an autosampler, a column heater, and fluorescence detector (Waters 2475 Fluorescence Multi) was used. Tryptophan analysis was performed separately, following the method of Sánchez-Machado et al. [21]. In summary, basic hydrolysis of proteins hydrolysates was performed in sodium hydroxide 4.2 M for 4 h at 120°C. Then, pH was adjusted to 9 with concentrated hydrochloric acid. The excitation wavelength was set at 280 nm and the emission at 348 nm. The column used is an Inertsil ODS-4150 mm × 4.6 mm, 5-micron particles (GL Sciences, Tokyo, Japan, provided by Canadian Life Science, Ontario, Canada). The isocratic elution system consisted of a mobile phase of 40 mM sodium acetate : methanol 80 : 20 (v/v) and a flow rate of 0.8 mL/minute.

### 2.5. Myotoxin Injury Protocol and Muscle Collection

At day 28 of feeding (day 0 after injury), one tibialis anterior (TA) muscle (32 animals per group) was chemically injured with bupivacaine. Buprenorphine (0.1 mg/kg), as an analgesic, was first administrated intraperitoneally, and then animals were anesthetized with inhaled isoflurane (2.5%/L O_2_). Anterior side of both TA muscles was shaved and disinfected with isopropyl alcohol before muscle injury. As previously reported [3], muscle injury was induced with 200 *μ*L of bupivacaine (0.5%) (Marcaine, Abbott, Mississauga, Ontario, Canada) injected at three sites—proximal, half proximal, and distal regions—within the TA using a syringe with a 29 G needle. The contralateral TA was injected with a similar volume of saline and served as control postinjury or noninjured muscle. The relevance of choosing the polypeptidic snake-venom bupivacaine is that, though causing acute muscle damage, this model preserves satellite cells and the basal lamina as well as the blood vessels and nerves [22]. After regaining self-consciousness, animals were returned to their cages and subjected to daily examinations for general health conditions; intraperitoneal buprenorphine (0.1 mg/kg) was also given twice daily until the third day after injury. Eight animals per group were not injured and used as baseline (preinjury control, day 0). On days 0, 2, 5, 14, and 28 after injury, both injured and noninjured TA were carefully removed (8 animals randomly selected from each group/time point) under anesthesia with isoflurane (2.5%/L O_2_) and processed to evaluate protein effects on inflammatory cell accumulation and muscle regeneration. Animals were then euthanized by cardiac exsanguination under anesthesia with inhaled isoflurane (2.5%/L O_2_) (PPC, Richmond Hill, Ontario, Canada) using an Ohmeda Isotec 3 Vaporiser (BOC Health Care, England).

As previously reported [3], muscle damage induced by bupivacaine involves an inflammatory response dominated by sequential accumulation of neutrophils and macrophages at time points selected in the current study. Therefore, days 2, 5, 14, and 28 were first selected to highlight the effect of dietary proteins on inflammatory cell (neutrophils and macrophages) accumulation at injured sites. It is known that neutrophil concentration, starting in few hours after injury, can remain at high concentrations until 5 days, while peak of ED1^+^-macrophages is generally observed at 5–7 days after injury and may remain high for several days [23]. In parallel to the increase of ED1^+^-macrophages the accumulation of anti-inflammatory ED2^+^-macrophages occurs and the initiation of skeletal muscle repair starts [3, 24]. In addition to the inflammatory response, muscle regeneration was evaluated as early as possible (days 2 and 5) and at intervals corresponding to progressive (days 5 and 14) and complete (day 28) muscle regeneration [3, 25].

### 2.6. Sample Preparation

TA muscles were weighed; mass was normalized to body weight at corresponding time points. Muscles were transversely cut in half across the midbelly section. Halves were either coated with cutting medium, rapidly frozen in liquid nitrogen-cooled isopentane, and stored at −80°C (DLT-21V-85ABA Harris, Massachusetts, USA) until immunochemistry, along with histomorphometry analyses, or lysed and centrifuged (10000 g, 10 min, 4°C), and the protein suspension was retained, aliquoted, and stored for ELISA and Western blot processing.

### 2.7. Immunohistochemistry and Cell Counting

10 *μ*m thick muscle sections adhered on coated slides were blocked with blocking buffer for 1 h. Sections were then incubated for 2 h at room temperature with primary antibody consisting of either mouse anti-rat W3/13 (CD43) (Serotec, Raleigh, North Carolina, USA, 1 : 50) to identify neutrophils, mouse anti-rat ED1 (CD68) (Serotec, Raleigh, North Carolina, USA, 1 : 100) to identify ED1^+^-macrophages, or mouse anti-rat ED2 (CD163) (Serotec, Raleigh, North Carolina, USA, 1 : 100) to identify ED2^+^-macrophages. Under sterile skeletal muscle injury, CD43 is neutrophil specific because eosinophils, basophils, monocytes, and B- and T-lymphocytes are not present. In mice, CD163 marker is specific to M2c macrophages; that subset closely resembles CD163-expressing macrophages in rats, known as ED2^+^ [26]. After washing with phosphate buffered saline (PBS), sections were incubated with anti-mouse IgG (Vector Laboratories, Burlington, Ontario, Canada, 1 : 200) for 1 hour at room temperature. Sections were then washed with PBS and incubated for 30 min with horseradish peroxidase avidin D (Vector Laboratories, Burlington, Ontario, Canada), after which they were revealed using diaminobenzidine chromogen (Cedarlane, Burlington, Ontario, Canada). After dehydration achieved by consecutive dipping in 75% ethanol, 95% ethanol, 100% ethanol, and xylene, sections were finally mounted under coverslips using Eukitt quick-hardening mounting medium (Sigma-Aldrich, Oakville, Ontario, Canada). Omission of primary antibody for one of the three sections on each slide served as a negative control. As previously reported [3, 9], labelled cells were viewed and counted in two nonoverlapping areas of each section through a 10 × 10 ocular grid at 400x magnification. A numbered grid divided into 100 squares was used to count inflammatory cells. The grid was initially set in the lower right of the section and was systematically moved up one grip until reaching the upper limit. The area of this grid was 0.0625 mm^2^ at 400x magnification. Counts were tightly reproducible since variations among 3 repeat counts were less than 1% for the same observer and less than 5% for different observers; cell profile for a given time point was counted by the same observer.

### 2.8. Histomorphometry

Cross-sectional area, percentage of centrally nucleated fibers, interstitial area (IA), and recovery index of regenerating muscles were evaluated as previously described [3]. In brief, three cross-sections (10 *μ*m) from the midportion of each frozen part of TA were stained with hematoxylin-eosin (Sigma-Aldrich, Oakville, Ontario, Canada), and two noncrossing images of each section were photographed at 200x magnification. Using Image J analysis software (Image J, National Institutes of Health, Maryland, USA), individual myofiber cross-sectional area (MCSA) was measured and summed to determine the total myofiber cross-sectional area (TMCSA). Total cross-sectional area (TCSA) was then traced and the IA was calculated by subtracting TMCSA from TCSA. As a good morphological index of fibers undergoing regeneration, central nucleation was also determined and expressed as a percentage of centrally nucleated fibers to total fibers for a given image. On average, MCSA was determined for 75 fibers per section, totalizing more than 200 fibers per muscle. The recovery index is the ratio of the average TA mass in the injured leg to the corresponding values for noninjured muscle [3].

### 2.9. Total Protein Content and Western Blot Assay

Muscle protein content was quantified using the BCA Assay Kit (Thermo Scientific, Mississauga, Ontario, Canada), which was standardized against bovine serum albumin according to the manufacturer's protocol. Protein suspension (50 *μ*g per well) was diluted in sample buffer, heated (~100°C) for 3 min, loaded into a 10% sodium dodecyl sulphate-polyacrylamide gel, and electrotransferred to Immobilon-P Transfer Membranes (Sigma-Aldrich, Oakville, Ontario, Canada), along with 5 *μ*L of Precision Plus Protein Dual Color Standards (Bio-Rad Laboratories, Ontario, Canada). Membranes were stained with Ponseau S to confirm protein transfer, after which they were serially washed with buffer, blocked with 5% BSA for 2 h, and then immunoblotted overnight at 4°C with either MyoD (C-20 : sc-304, 1 : 500) or myogenin (M-225 : sc-576, 1 : 200) polyclonal rabbit IgG as primary antibody (Santa Cruz Biotechnology, Santa Cruz, California, USA), all diluted in 3% BSA. As previously reported [3], levels of MyoD and myogenin were measured at day 2 and day 5 after injury, respectively. As opposed to MyoD which is a marker of satellite cell activation upregulated by day 2 after injury, myogenin is highly expressed when differentiation and myotube formation are initiated from day 3 to day 5 after injury [5]. Membranes were washed and incubated with HRP-linked-mouse anti-rabbit IgG polyclonal secondary antibody (sc-2357) (Santa Cruz Biotechnology, Santa Cruz, California, USA) at a dilution of 1 : 10 000 in BSA (3%) for 1 h at room temperature. Bands were revealed using ECL-plus Western blotting reagent (PerkinElmer Life and Analytical Sciences, Wellesley, Massachusetts, USA) according to manufacturer's instructions. The signal intensities were captured (Fusion FX7, Montreal Biotech Inc., Montreal, Canada), corrected for local background, and quantified using BIO-1D advanced software (Montreal Biotech Inc., Montreal, Canada). Optical densities were normalized to GAPDH (Santa Cruz Biotechnology, Santa Cruz, California, USA).

### 2.10. Statistical Analysis

Data was analysed with the MIXED procedure of the Statistical Analysis System (SAS Institute, version 9.2, Cary, North Carolina, USA). Normality was tested according to Shapiro-Wilk test and observed for all data, except for myogenin which was log-transformed. Diet effects nested within each time point (days 0, 2, 5, 14, and 28 after injury) were determined with an analysis of variance (ANOVA); Fisher's protected LSD* post hoc* test was used to evaluate significance. Power calculation at 80% from data published by our research team [3] showed that a sample size of 7 rats per dietary group per time point was determined based on the efficacy of dietary cod protein at influencing muscle weight and inflammatory response at a probability level inferior to 0.05. One outlier was removed from statistical analysis of most measured parameters (centrally nucleated fibers, muscle mass, MCSA, interstitial area, density of neutrophils, and density of ED1^+^-macrophage and ED2^+^-macrophages); therefore values are presented for *n* = 7-8 rats/group/time point.

## 3. Results

### 3.1. Food Intake and Body Weight Gain

Initial body weight was similar between groups (Figure 1(b)). Altering dietary protein source did not affect daily mean food intake (Figure 1(c)), final body weight (Figure 1(d)), nor body weight gain (Figure 1(e)) during all the experimental period.

### 3.2. Morphometric Assessment of Muscle Regeneration

**Figure 2:**
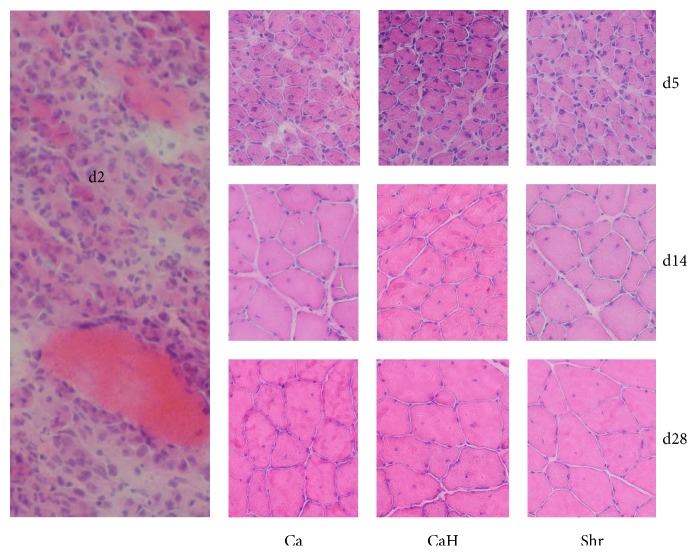
Representative H/E stained cross-sections from injured tibialis anterior muscle.

At day 2 after injection, bupivacaine destroyed a large proportion of muscle fibers in all groups (Figure 2). However, as a result of the onset of muscle regeneration observed at day 5 after injury, necrotic fibers were progressively replaced by regenerating and growing fibers with high proportion of central nuclei (Figure 2). At day 28 after injury, the proportion of central nucleation was similar to that observed at day 14 after injury and was not influenced by the diets.

**Figure 3:**
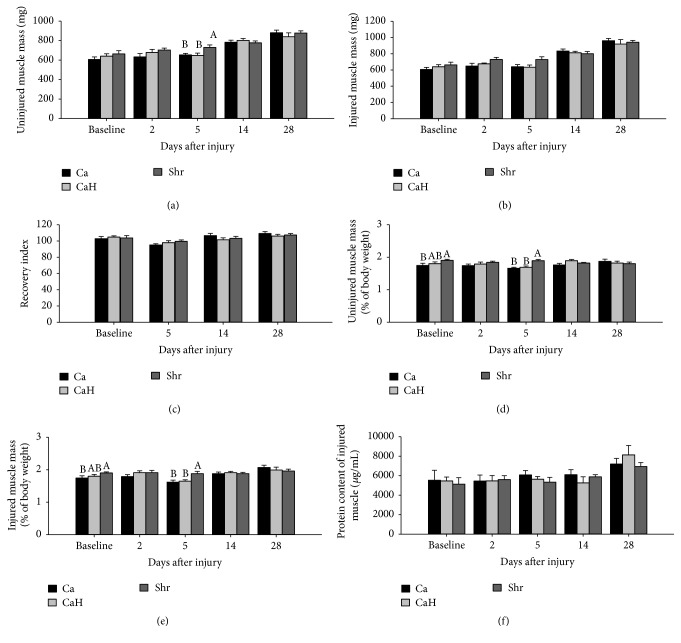
Muscle mass recovery of regenerating tibialis anterior muscle in rats. Noninjured and bupivacaine-injured muscles were collected at days 0, 2, 5, 14, and 28 after injury, weighed, and normalized to body weight. Absolute muscle weights are presented for noninjured (a) and injured (b) muscles. A recovery index, as the percentage of injured values relative to noninjured values, was calculated and presented for muscle mass (c). Muscle mass is also presented as a percentage of body weight for injured (d) and noninjured (e) muscles. BCA Assay Kit was used to determine protein content in injured muscles (f). Values are mean ± SEM (*n* = 7-8 rats per group/time point). Groups bearing different letters for a given time point are significantly different (*p* ≤ 0.05). Ca, casein; CaH, casein hydrolysate; Shr, shrimp protein hydrolysate.

Absolute muscle mass in both noninjured (Figure 3(a)) and injured (Figure 3(b)) muscles did not change with treatments at any studied time points, except for day 5 after injury where Shr significantly increased TA mass in noninjured muscle (Shr versus Ca, *p* = 0.015; Shr versus CaH, *p* = 0.014), while it tended to increase injured muscle mass compared with either Ca (*p* = 0.070) or CaH (*p* = 0.060). The recovery index was not affected by diets and remained unchanged across time point (Figure 3(c)). When expressed relative to body weight (Figures 3(d), 3(e), and 3(f)), the positive effect of Shr on muscle mass was clearly visible at day 0 and day 5 after injury compared with Ca in both noninjured (day 0, *p* = 0.004; day 5, *p* = 0.002) and injured (day 5, *p* = 0.018) muscles. Higher muscle mass was also observed in Shr-fed animals when compared with CaH-fed group at day 5 after injury in both noninjured (*p* = 0.007, Figure 3(d)) and injured (*p* = 0.038, Figure 3(e)) muscles. Prior to injury, muscle mass in CaH-fed rats was intermediary to, and not significantly different from, that of Ca- and Shr-fed counterparts (Figures 3(d) and 3(e)). No difference was observed at days 2, 14, and 28 after injury between groups (Figures 3(d) and 3(e)). In contrast to muscle mass, TA protein content measured in injured legs did not change in response to protein feeding at any time point (Figure 3(f)).

**Figure 4:**
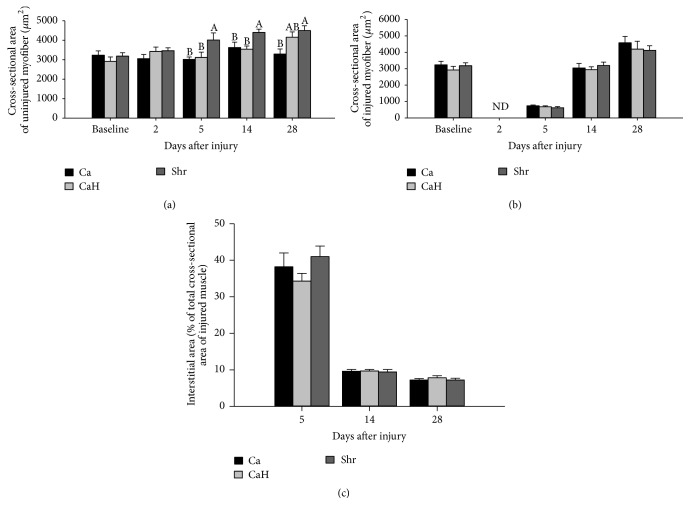
Muscle fiber cross-sectional area and interstitial area (%TCSA) of regenerating tibialis anterior muscle in rat. Myofiber cross-sectional area (MCSA) of three H/E-stained cryosections was quantified at days 0, 5, 14, and 28 after injury. Graphs are presented for noninjured (a) and injured (b) muscles. Two images were captured at 200x magnification from each cryosection and MCSA was traced using Image J analysis software. Total cross-sectional area (TCSA) was then traced in the injured muscle, and the interstitial area (IA) was quantified by subtracting the sum of individual MCSA from the TCSA at days 5, 14, and 28 after injury (c). Values are expressed as mean ± SEM (*n* = 7-8 rats per group/time point). Groups bearing different letters for a given time point are significantly different (*p* ≤ 0.05). ND, nondetermined because of massive destruction of the myofibers in the necrotic area. Ca, casein; CaH, casein hydrolysate; Shr, shrimp protein hydrolysate.

Figure 4 shows MCSA in both noninjured (Figure 4(a)) and injured muscles (Figure 4(b)) and interstitial area in the injured TA (Figure 4(c)). Shr significantly increased MCSA in noninjured muscle at days 5 and 14 after injury compared to either Ca (day 5, *p* = 0.009; day 14, *p* = 0.027) or CaH (day 5, *p* = 0.016; day 14, *p* = 0.014) (Figure 4(a)). At day 28 after injury, while MCSA in noninjured muscles remained higher in the Shr group compared with the Ca group (*p* = 0.008), values for CaH matched levels observed in the Shr group and strongly tended to be higher from that of the Ca group (*p* = 0.051) (Figure 4(b)). Starting approximately at 3000 *μ*m^2^ prior to muscle injury, MCSA values in injured muscles drastically diminished at day 5 after injury and then increased to baseline level at day 14 after injury (day 5 versus day 14, *p* < 0.0001; day 0 versus day 14, *p* = 0.993), while continuing to increase until day 28 after injury (*p* < 0.0001) (Figure 4(b)). As a consequence of necrotizing effects of bupivacaine, MCSA was barely null in the injured muscle at day 2 after injury (Figure 4(b)). At these time points, MCSA in both noninjured and injured muscles was closely similar between Ca and CaH (Figures 4(a) and 4(b)). In relation to the lack of effect on regenerating MCSA, diets had no impact on interstitial area of regenerating TA (Figure 4(c)).

### 3.3. Effect of Shrimp Protein Hydrolysate on the Timing of Inflammatory Cells

**Figure 5:**
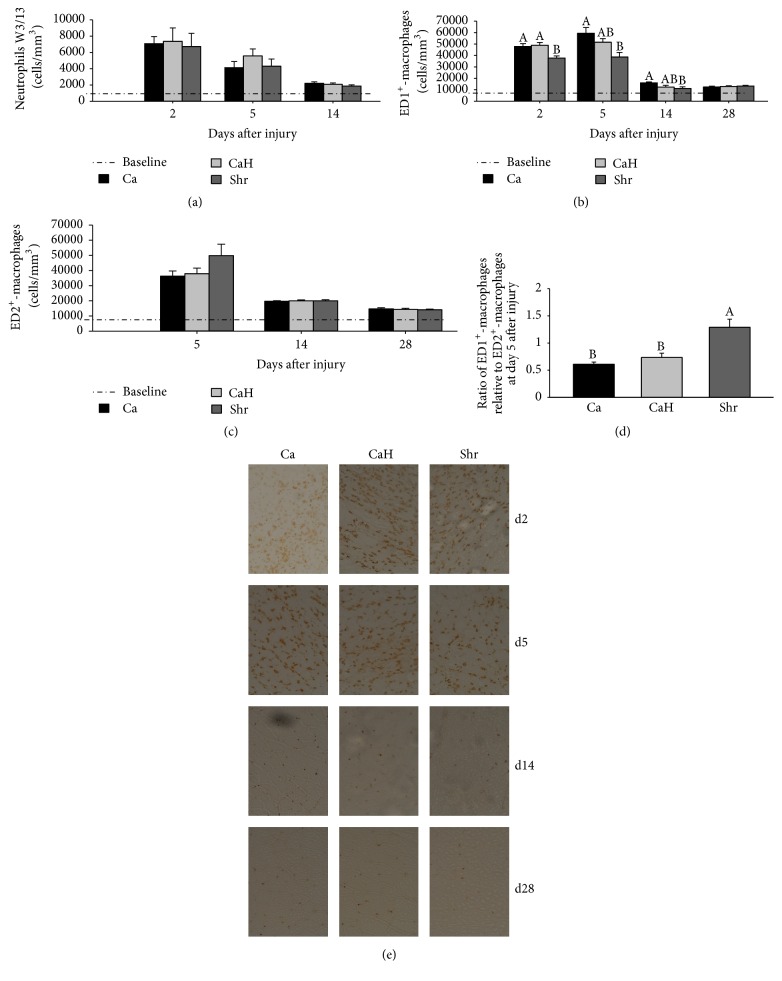
Time course of inflammatory cell accumulation in rat tibialis anterior muscle following bupivacaine injection. Transverse sections (10 *μ*m) were immunoassayed with specific antibodies against neutrophils (W3/13) (a), ED1^+^-macrophages (b), or ED2^+^-macrophages (c). (d) shows the ratio of ED2^+^ relative to ED1^+^-cells at day 5 after injury. Labelled cells were counted at 400x magnification and expressed as a number of cells/mm^3^. Immunostained sections for ED1^+^-macrophages are also presented in (e); labelled ED1^+^-macrophages are identified by brown dots (e). Values are mean ± SEM (*n* = 7-8 rats per group/time point). Groups bearing different letters for a given time point are significantly different (*p* ≤ 0.05). Ca, casein; CaH, casein hydrolysate; Shr, shrimp protein hydrolysate.

The timing of neutrophils and ED1^+^- and ED2^+^-macrophages are given in Figures 5(a), 5(b), and 5(c), respectively. Irrespective of the group, days 2 and 5 after injury were characterized by a high number of neutrophils and macrophages, which progressively decreased to basal level by day 14 after injury (Figures 5(a), 5(b), and 5(c)). While no effect of protein feeding was observed on neutrophil density (Figure 5(a)), Shr significantly reduced ED1^+^-macrophages compared with either Ca (*p* = 0.013) or CaH (*p* = 0.006) at day 2 after injury (Figures 5(b) and 5(d)). Similar response was observed for Ca and CaH at that time. At days 5 and 14 after injury, ED1^+^-macrophages were lower in injured muscles of Shr-fed rats compared with Ca counterparts (day 5, *p* = 0.006; day 14, *p* = 0.038), while no difference was observed when compared with CaH, which was not significantly different from Ca. By day 14 after injury, ED1^+^-macrophages returned to basal level in Shr and CaH. Such a positive effect was observed in the Ca group only at day 28 after injury. While no diet effect was observed on ED2^+^-macrophages (Figure 5(c)), the ratio of ED2^+^-cells relative to ED1^+^-cells at day 5 after injury was higher in Shr-fed group as compared to either Ca- (Ca versus Shr, *p* = 0.0002) or CaH- (CaH versus Shr, *p* = 0.008) fed counterparts (Figure 5(d)).

### 3.4. Effect of Dietary Proteins on MyoD and Myogenin Content

**Figure 6:**
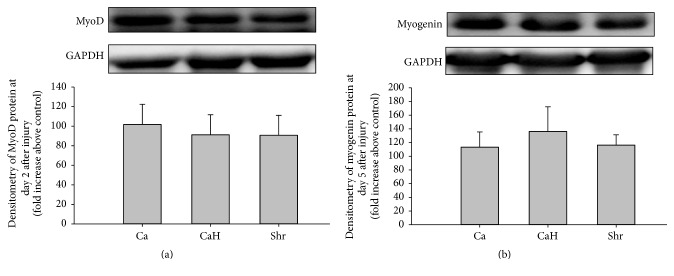
MyoD and myogenin proteins in regenerating muscles. Expression of MyoD (a) and myogenin (b) (both are usually upregulated during muscle regeneration) was measured by immunoblotting in regenerating muscles at day 2 and day 5, respectively. A representative blot is displayed in the upper panel for each. All values were corrected for GAPDH as a protein loading control. Results are expressed as a percentage of values obtained at day 0 (mean optical density ± SEM, *n* = 7-8/group). Ca, casein; CaH, casein hydrolysate; Shr, shrimp protein hydrolysate.

As protein feeding did not impact regenerating MCSA, no significant difference was observed neither for the myogenic regulatory factor MyoD measured at day 2 after injury (Figure 6(a)) nor for the differentiation myogenin quantified at day 5 after injury (Figure 6(b)).

## 4. Discussion

The current study focused specifically on the effect of feeding shrimp protein hydrolysate on neutrophil and macrophage accumulation in bupivacaine-injured TA that is prone to mimicking exercise-induced muscle injuries. Particular strengths of that experimental approach are robustness and repeatability since magnitude of results reported here are closely similar to that previously achieved by our group using the same protocol in rats [3]. The main finding of the present work is that shrimp hydrolysate reduced injury-induced inflammation through deceasing ED1^+^-macrophages when compared with casein; while increasing MCSA in noninjured muscles, shrimp hydrolysate failed to impact corresponding injured muscles. We thus conclude that feeding shrimp protein hydrolysate can specifically modulate inflammation without allowing optimal recovery from damage in terms of muscle mass and MCSA but enhances fiber growth in noninjured muscles. On the other hand, the beneficial effect of shrimp hydrolysate on ED1^+^-cells was mostly mimicked by the casein hydrolysate at day 5 and day 14 after injury, suggesting that the hydrolysis process might be an additional way to enhance the anti-inflammatory action of dietary proteins.

As a result of bupivacaine injection, damaged muscle fibers were massively invaded by neutrophils and macrophages, as previously reported [3]. One key observation of the present work is that Shr modulated inflammation through reducing ED1^+^-cells compared with Ca and CaH at day 2 after injury; peak ED1^+^-cells occurring on days 2 and 5 after injury had returned to baseline values by day 14 after injury in the Shr group. Baseline level of ED1^+^-cells was observed in the Ca group in a longer term—by day 28 after injury—indicating faster resolution of inflammation in the Shr group. Turning attention to muscle regeneration has pointed the timing of macrophage resolution as key player to this process [27, 28]. Macrophages are major inflammatory cells recruited into injured muscles that display remarkable plasticity. They are immunologically classified into two main subsets according to their function and environmental cues [29]: “classically activated M1 macrophages” are present in the inflammatory period and associated with phagocytosis, while the second wave of “alternatively activated M2 macrophages” follows once necrotic tissue has been removed and actively participates in the regeneration and remodeling processes. M1-M2 classification specifically applies to mouse macrophages whereas the corresponding ED1-ED2 classification applies to rat macrophage subtypes. Like neutrophils, accumulation of phagocytic ED1^+^-cells can promote muscle damage via cytotoxic levels of proinflammatory factors (TNF-*α*, IL-1*β*, IL-6, PGE-2, etc.) and free radical-mediated mechanisms [2], impairing regeneration. In support of this, ED1^+^-cells density was negatively correlated with injured muscle mass, particularly at day 5 after injury that characterized the initiation of muscle regeneration (*r* = −44, *p* = 0.0436, and *n* = 21). However, predominance of anti-inflammatory ED2^+^ phenotype is known to stimulate satellite cell proliferation and differentiation through releasing growth factors and promoting resolution of inflammation [27, 30]. Although ED2^+^-cell count was not significantly altered by protein feeding, ratios of ED2^+^-cells relative to ED1^+^-cells at day 5 after injury were 0.61 ± 0.04, 0.80 ± 0.08, and 1.32 ± 0.15 in Ca (Ca versus Shr, *p* = 0.0002), CaH (CaH versus Shr, *p* = 0.008), and Shr groups, respectively, suggesting an accelerated switch of proinflammatory ED1^+^-cells toward the anti-inflammatory phenotype (ED2^+^) in the Shr group [27, 31–33]. In close agreement with the present study, convincing findings from our lab have shown that cod protein feeding reduced ED1^+^-cells up to 22% at days 2, 5, and 14 in rat tibialis anterior muscle injected with bupivacaine, through its high levels of arginine, glycine, and taurine [3]. A casein-based diet enriched with arginine, glycine, taurine, and lysine, to match their respective level in cod protein, reduced ED^1+^-cells in a similar way as did cod protein compared with casein alone [3]. In accordance with our hypothesis, it is therefore likely that faster ED1^+^-cell resolution in Shr-fed animals is related to higher content of arginine and glycine, since Shr had 116% and 150% more arginine and glycine than Ca, respectively (Table 1). In a similar way, Shr provided 188% and 217% more arginine and glycine than CaH, respectively. Anti-inflammatory actions of either arginine or glycine could include slower recruitment of leukocytes to the injury site, owing to their capacity to inhibit chemotaxis, leukocyte rolling, and transmigrating out of the vessels [34–37]. In this respect, it has been shown that either arginine or glycine reduced macrophage accumulation in renal allografts [38] and rodent postoperative ileus [37], respectively. Through decreasing proinflammatory cytokines translated into the NF-*κ*B pathway [14, 15, 18], high levels of arginine and glycine in Shr might have attenuated chemotactic recruitment of new leukocytes, resulting in lower ED1^+^-cells. Moreover, it is also possible that, through the hydrolysis process [8], generation of unidentified peptides displaying biological activity might improve anti-inflammatory properties of both Shr and CaH; that concept is supported by the fact that CaH closely mimicked the ED1^+^-cell response seen with Shr at days 5 and 14 after injury, while Ca took a longer time. Calcitonin-gene-related peptide and calcitonin were found to prevent macrophage activation [39]. It is thus possible that calcitonin present in the shrimp protein source used here could partly explain its anti-inflammatory property [10]. However, whether decreased proinflammatory cytokines occur in direct relation to reduced ED1^+^-cells in response to Shr feeding will require further investigations.

Despite having beneficial effects on inflammation, Shr enhanced muscle growth rather than improve fiber recovery compared with either Ca or CaH. A study by Dort et al. [3] on bupivacaine-injured muscles showed that the regeneration process, assessed by larger regenerating, centrally nucleated fibers and increased level of myogenin, was clearly present by day 5 after injury in rats consuming cod protein compared with casein. Such positive effects of cod protein were associated with higher ED2^+^-macrophage accumulation [3], implying thus greater local release of growth factors for the onset of muscle regeneration. Therefore, because Shr failed to have an ED2^+^ response, local release of growth factors might have been limited, thus not allowing optimal recovery. Moreover, it is known that injury as well as age-related atrophy can cause a blockage in IGF-1/Akt-mediated signaling during muscle recovery [3, 40]. Thus, further studies to determine whether Shr can impact IGF-1-Akt regulating atrophic and hypertrophic effectors during muscle recovery from injury are needed.

In contrast to fiber recovery, Shr feeding resulted in greater muscle growth, a finding in line with our previous observation that cod protein increased fiber growth when compared with casein [3]. More importantly, the fact that higher MCSA was observed in rats that received Shr compared with Ca while both Shr and Ca had similar noninjured muscle mass suggests increased protein synthesis and lower fat accumulation within noninjured muscles of Shr-fed rats. In this respect, it has been demonstrated that casein-fed rats exhibited a decrease in body “protein : fat” ratio compared with their counterparts fed a marine-derived protein [41]. These findings are likely to be related to higher content of essential amino acids, in particular isoleucine, lysine, and threonine, in Shr compared with either Ca or CaH (Table 1). In the present study, while containing up to 8% isoleucine, 25% lysine, and 27% methionine more than CaH, Shr provided 34%, 12%, and 90% more isoleucine, lysine, and methionine than CaH, respectively (Table 1). Higher supply of essential amino acids might thus result in enhanced anabolic potential of Shr [42–44], thereby improving growth of muscle fibers. However, it is worth noting that CaH closely reproduced the enhanced fiber growth seen with Shr at the end of the study period, although MCSA values for the Ca group remained lower. This finding strongly suggests beneficial effects of the hydrolysis process through generating biological peptides as well as fostering faster availability of amino acids, allowing optimal postprandial protein accretion in CaH compared with intact Ca known as a slow protein [45].

A limitation of this study is that some parameters were presented only at what was seen to be the most relevant time points according to previous studies [3, 23–25, 46]. It is possible that including other time points—such as day 3 after injury for MyoD measurement—would be more appropriate to observe differences between dietary proteins. Because serum inflammatory markers (TNF-*α* and IL-6) were not detected at the same studied time points [3], sampling at other time points (e.g., ≤24 h after injury) would be helpful in detecting cytokines changes in response to dietary protein feeding. Shellfish is an excellent natural source of taurine, and very high levels are typically found in clams, scallops, and shrimp [47]. Because taurine is heat-sensitive, it might be destroyed during the production of the Shr hydrolysate, which was mechanically extracted from by-products of shrimp processing plants (Merinov Centre, Quebec, Canada). Moreover, including a group fed with a nonhydrolyzed shrimp protein would add further support to the beneficial effect seen with shrimp protein and allow a more direct comparison with the cod protein previously used [3]. Determining specific peptides or amino acids underlying the effect of shrimp protein was also beyond the scope of the current study.

To summarize, this study provided consistent evidences of improved resolution of inflammation in chemically injured rat skeletal muscles after consumption of a marine-derived protein, specifically shrimp protein hydrolysate. Shr mainly blunted ED1^+^-macrophage accumulation during the recovery process compared with Ca, while allowing early infiltration of such inflammatory cells at the site of injury. Further discoveries of potential mechanisms of actions underlying the beneficial effect of Shr, in addition of cytokine assessment, will advance our understanding on the integral role of Shr in inflammation in order to provide a widely applicable alternative against injury-induced muscle inflammation.

## Figures and Tables

**Table 1:** Amino acid composition of dietary protein sources (g/100 g of amino acids).

Amino acids	Dietary treatments		
	Ca	CaH	Shr
Alanine	2.83	3.46	5.59
Arginine	2.90	2.18	6.28
Aspartic acid	6.17	5.85	9.97
Cysteine	4.62	5.96	0.79
Glutamic acid	15.97	12.22	12.40
Glycine	1.88	1.48	4.70
Histidine	2.49	1.45	2.61
Isoleucine	3.70	2.98	4.01
Leucine	7.63	6.57	7.00
Lysine	6.07	6.77	7.62
Methionine	2.34	1.97	2.05
Phenylalanine	4.90	2.90	4.24
Proline	9.93	7.51	3.89
Serine	4.45	2.41	4.00
Threonine	3.13	2.10	3.99
Tryptophan	1.00	1.05	0.84
Tyrosine	4.57	3.15	3.66
Valine	4.60	3.80	4.22
EAA^1^	35.86	29.59	36.6

Ca, casein; CaH, casein hydrolysate; Shr, shrimp hydrolysate.

^1^Sum of essential amino acids (histidine, isoleucine, leucine, methionine, lysine, valine, threonine, phenylalanine, and tryptophan).

**Table 2:** Composition of the purified diets (g/100 g of diet).

Ingredients	Dietary treatments		
	Ca	CaH	Shr
Sucrose^1^	20	20	20
Cellulose^1^	5.69	5.69	5.69
Cornstarch^1^	32.1	32.1	33
Casein^1^	22.2	—	—
Casein hydrolysate^1^	—	22.2	—
Shrimp hydrolysate^2^	—	—	21.3
Cholesterol^1^	1	1	1
Lard^3^	10	10	10
Soya oil^4^	4	4	4
Minerals^1,5^	3.5	3.5	3.5
Vitamins^1,6^	1	1	1
BHT^1^	0.2	0.2	0.2
Choline bitartrate^1^	0.3	0.3	0.3

Ca, casein; CaH, casein hydrolysate; Shr, shrimp hydrolysate; BHT, butylated hydroxytoluene.

^1^Purchased from MP Biochemicals (Solon, Ontario, Canada).

^2^Shrimp hydrolysate from Merinov (Quebec, Canada).

^3^Purchased from local supermarket (Maple Leaf, Burlington, Canada).

^4^Purchased from local supermarket (Loblaws Inc., Toronto, Ontario, Canada).

^5^AIN-93G purified mineral mix for laboratory rodents (product number: 02960400). AIN-93G mineral mix provides the following (g/100 g mix): calcium carbonate, 35.7; monopotassium phosphate, 19.6; potassium citrate monohydrate, 7.078; sodium chloride, 7.4; potassium sulphate, 4.66; magnesium oxide, 2.4; ferric citrate, 0.606; zinc carbonate, 0.165; manganese carbonate, 0.063; copper carbonate, 0.03; potassium iodate, 0.001; sodium selenate anhydrous, 0.00103; ammonium molybdate *∗* 4H_2_O, 0.000795; sodium metasilicate *∗* 9H_2_O, 0.1454; chromium potassium sulphate *∗* 12H_2_O, 0.0275; lithium chloride, 0.00174; boric acid, 0.008145; sodium fluoride, 0.00635; nickel carbonate, 0.00318; ammonium vanadate, 0.00066; powdered sugar, 22.1.

^6^AIN-93 VX Vitamin Mix Fortification provides the following (g/kg mix; product number: 0296040201): nicotinic acid, 3.0; D-calcium pantothenate, 1.6; pyridoxine hydrochloride, 0.7; thiamine hydrochloride, 0.6; riboflavin, 0.6; folic acid, 0.2; d-biotin, 0.02; vitamin B_12_ (0.1% triturated in mannitol), 2.5; *α*-tocopherol powder (250 U/g; 184 mg/g), 300.0; vitamin A palmitate (250 000 U/g; 137 mg/g), 1.6; vitamin D_3_ (400 000 U/g; 10 000 mg/g), 0.25; phylloquinone, 0.075; powdered sucrose, 959.655.
